# Impact of the Interaction Between Screen Time and Activity Interests on Adolescent Depression Risk: Construction of a Predictive Model Based on Machine Learning

**DOI:** 10.62641/aep.v54i2.2068

**Published:** 2026-04-15

**Authors:** Leiming Mao, Ruiqi He, Shike Zhang, Yongqian Ge, Entong Xu, Yalan Chen, Gujun Cong, Haiyan Miao, Yunjie Jiang, Haijiao Zhu

**Affiliations:** ^1^Nantong Mental Health Center, 226001 Nantong, Jiangsu, China; ^2^Department of Medical Informatics, School of Medicine, Nantong University, 226001 Nantong, Jiangsu, China; ^3^Department of Radiology, Affiliated Hospital of Nantong University, 226001 Nantong, Jiangsu, China

**Keywords:** depressive disorder, adolescent, machine learning, screen time, leisure activities, risk assessment

## Abstract

**Background:**

Adolescent depression is an increasing public health concern, with excessive screen time elevating depression risk and activity interests providing protection. However, most studies examine these behaviors separately and rely on limited analytical methods. This study used machine learning (ML) to develop a predictive model and evaluate the combined influence of screen time and activity interests on adolescent mental health.

**Methods:**

A multi-center survey was conducted among adolescents aged 10–14 years in Chongchuan District, Nantong. Depression-related domains were assessed using the Child and Adolescent Mental Health Screening Questionnaire, integrating seven validated scales. A two-stage feature-selection strategy identified 11 key predictors. Three ML models (logistic regression [LR], extreme gradient boosting [XGBoost], and categorical boosting [CatBoost]) were trained with an 80:20 stratified split. Class imbalance was addressed using synthetic minority over-sampling technique and class-weighting. Model performance and interpretability were evaluated using receiver operating characteristic (ROC) and calibration curves, partial dependence plots, and shapley additive explanations (SHAP) analyses.

**Results:**

A total of 2202 valid questionnaires were analyzed. The distribution of depression severity was as follows: safe 59%, mild 17%, moderate 11%, and severe 13%. The integrated questionnaire demonstrated strong reliability (Cronbach’s α = 0.910) and good construct validity (Kaiser–Meyer–Olkin [KMO] = 0.91; root mean square error of approximation [RMSEA] = 0.049; and comparative fit index [CFI] = 0.859). ROC-Youden analysis confirmed expert-defined cutoffs (29, 32, and 35). Feature selection identified 11 key predictors, with activity interest and psychological functioning consistently ranking highest in importance. Across the three ML models, LR exhibited the best generalizability, XGBoost showed overfitting, and CatBoost achieved balanced performance. SHAP and partial dependence analyses revealed nonlinear screen-time effects and dose-dependent protective effects of activity interest, including the moderation of high screen exposure in severe-risk groups.

**Conclusions:**

This study suggests that ML models can be used to screen adolescents at risk of depression by capturing the combined influence of screen time and activity interests. The model is intended for screening rather than diagnosis and may support school-based early identification, and further validation in clinical contexts is needed.

## Introduction

Depression is a prevalent global mental health disorder that profoundly affects emotional, cognitive, behavioral, and physiological functioning. According to a 2023 report from the World Health Organization [1], depression affects approximately 3.8% of the global population and contributes to nearly 700,000 suicide deaths each year. Recent data from China’s National Health Commission (2024) [2] estimate a 2% prevalence among adolescents, accompanied by marked impairments in social functioning, academic performance, and overall quality of life. In the diagnostic and statistical manual of mental disorders, fifth edition (DSM-5), depression is characterized by persistent low mood and anhedonia, often accompanied by impaired concentration, sleep disturbances, hopelessness, and reduced self-worth.

Growing evidence supports the involvement of important neurobiological mechanisms in the association between modern behaviors and depression [3]. Disruptions in circadian rhythms and melatonin secretion, often exacerbated by blue-light (BL) exposure from digital screens, are strongly associated with sleep disturbances, a core symptom of depression. Screen-emitted BL delays melatonin release, prolongs sleep latency, reduces REM sleep, and decreases morning alertness, creating a maladaptive sleep-wake cycle that may heighten vulnerability to depressive symptoms [4, 5]. Additionally, chronic stimulation of the reward system by digital media can dysregulate dopamine pathways, contributing to anhedonia by impairing the brain’s ability to process pleasure [6].

Therefore, screen time has emerged as a key behavioral factor in adolescent mental health research. Przybylski and Weinstein [7] demonstrated that more than 2 hours of daily screen exposure significantly reduces psychological adaptability in adolescents. Twenge *et al*. [8] further linked excessive social media use to increased depression risk, especially among girls. A synthesis of 35 longitudinal studies suggested that higher screen time in young people (aged 10–24 years) is associated with subsequent depressive symptoms, although the effect size was small [9]. Pre-sleep screen use [10] appears particularly detrimental, as it disrupts circadian rhythms and further impairs sleep quality [11], heightening depression risk.

Conversely, activity interests, including physical, artistic, and social activities, serve as protective psychological resources [12]. The study of Fredricks and Eccles [13] reported that adolescents with greater activity engagement exhibit better emotional well-being, greater self-efficacy, stronger social connectedness, and enhanced resilience. In contrast, lack of interest or displacement by excessive entertainment media [3] (e.g., short video and gaming) increases social withdrawal and negative affect, elevating depression vulnerability.

Despite extensive research on screen time and activity interests, current studies have four key limitations: (1) most examine these factors independently, overlooking their potential interaction; (2) traditional statistical approaches often fail to capture the nonlinear, multidimensional nature of these behaviors; (3) existing assessments rely on static questionnaire scores without dynamic predictive capacity; and (4) there is a paucity of evidence specific to Chinese adolescents, a population with distinct cultural and developmental digital habits.

By modeling nonlinear patterns and integrating complex behavioral features, machine learning (ML) [14] provides a promising methodological solution to these challenges. Prior studies, such as those by Razavi *et al*. [15], Randhavane *et al*. [16], and Jacobson *et al*. [17], demonstrate the effectiveness of ML in identifying risk markers using mobile metadata, affective emotional signals, and passive sensing data, behavioral indicators, and wearable sensors, offering a foundation for intelligent and personalized mental health assessment.

This study applied ML techniques to a large, school-based Chinese adolescent cohort to address the above research gaps. By integrating detailed screen use behaviors and activity interests, the study aimed to (1) develop a multi-level depression risk classification model, (2) investigate associations between screen exposure and activity engagement, and (3) establish a practical, scalable risk prediction tool suitable for early intervention in school settings. These techniques may enable more precise risk detection and inform tailored prevention strategies for adolescent depression.

## Materials and Methods

### Determination of Study Subjects

This study was conducted as part of the Mental Health Promotion Project for Children and Adolescents in Chongchuan District and utilized data from the 2024 Nantong Adolescent Mental Health Cohort, a longitudinal school-based study. The study was approved by the Medical Ethics Committee of the Fourth People’s Hospital of Nantong City, China (ethics approval number: 2022-k041). The present analysis included primary and junior high school students aged 10–14 years. A stratified cluster sampling method was employed. Two primary and two junior high schools were randomly selected (each having more than 10 classes per grade) from the 60 primary and junior high schools in the district. Within each selected school, five classes per grade (grades 4–9) were randomly sampled, and all students in these classes were invited to participate. This sampling strategy ensured representative coverage across educational stages and school types.

### Inclusion Criteria

The inclusion criteria were as follows. (1) Age stratification: Participants were required to be 10–14 years old (corresponding to early to mid-pubertal stages) to ensure developmental homogeneity. (2) Assessment competency: Participants needed to meet both of the following conditions: (i) ability to independently comprehend and complete Chinese-language questionnaires, and (ii) possess no clinically significant language, cognitive or developmental impairments that might compromise response validity.

### Exclusion Criteria

The exclusion criteria were as follows. (1) History of severe mental illness: Participants with professionally diagnosed psychotic disorders (e.g., schizophrenia spectrum disorders or bipolar disorder) were excluded. (2) Recent major traumatic events: Participants who had experienced major life stressors within the past month (e.g., bereavement or serious accidents) were excluded. (3) Poor questionnaire quality: Questionnaires were excluded if they contained: (i) ≥20% missing data, or (ii) clear logical inconsistencies (e.g., reporting “>2 hours daily video viewing” together with “no screen time”). (4) Severe physical illness: Participants with significant somatic conditions (e.g., active malignancies or epilepsy) that could confound mental health assessments were excluded.

### Development of the Child and Adolescent Mental Health Screening Questionnaire

The study employed the Child and Adolescent Mental Health Screening Questionnaire, developed by Nantong Mental Health Center (NMHC), as a multidimensional tool for assessing psychological and environmental factors related to adolescent depression. This instrument incorporates constructs from seven validated psychological scales: the Patient Health Questionnaire-9 (PHQ-9; depression symptoms), the 13-item Beck Depression Inventory (BDI-13; cognitive-affective symptoms), Egna Minnen Beträffande Uppfostran (EMBU; parenting styles), the Middle School Students Mental Health Scale (MSSMHS; general mental health), the Social Avoidance and Distress Scale (SADS; social avoidance and distress), the Pittsburgh Sleep Quality Index (PSQI; sleep quality), and the Internet Addiction Disorder Diagnostic Scale (IADDS; problematic internet use). The instrument was culturally adapted to a Chinese adolescent context while preserving the psychometric properties of the original instruments (**Supplementary Material 1**).

Items were selected based on theoretical relevance to harmonize these heterogeneous sources, converted to a unified 4-point Likert format, and reorganized into four functional subdomains: Depression Index, Stressful Events, Screen Use, and Sleep Status. The PHQ-9 and the BDI-13 contributed depressive and cognitive-affective symptoms (9 and 13 items; α ≈ 0.80–0.89 and >0.85). The EMBU, MSSMHS, and SADS provided indicators of parenting styles, psychological strain, and social adaptation (scores of 1–4; α ≈ 0.70–0.90). Sleep and behavioral regulation were assessed using the PSQI (19 items, scores of 0–21; α ≈ 0.70–0.83) and the IADDS (≈20 items; α ≈ 0.80–0.88).

After standardization, 43 items were retained for the final instrument. Psychometric testing showed excellent internal consistency (Cronbach’s α = 0.910), strong split-half reliability (Guttman = 0.865), and good structural validity (Kaiser–Meyer–Olkin [KMO] = 0.91, root mean square error of approximation [RMSEA] = 0.049, and comparative fit index [CFI] = 0.859), confirming that the integrated questionnaire provides a concise and reliable multidimensional measure of child and adolescent mental health.

### Item Selection and Construction of the 43-Item Core Questionnaire

The seven original scales contained more than 180 items. A three-stage item-selection procedure integrating theory-driven and data-driven criteria was applied to construct a concise and psychometrically coherent screening tool.

Stage 1: Theory-guided initial screening. All items were categorized into four conceptual domains informed by conceptual frameworks of adolescent depression, diagnostic criteria from the International Classification of Diseases, 11th Revision (ICD-11) and the Diagnostic and Statistical Manual of Mental Disorders, Fifth Edition (DSM-5), and constructs commonly used in school-based mental-health screening: Depression Index (emotional, cognitive, motivational, and somatic symptoms), Stressful Events (family, peer, school, and environmental stressors), Screen Use, and Sleep Status. Items unrelated to these constructs were excluded, resulting in 84 items.

Stage 2: Psychometric reduction. Exploratory and confirmatory factor analyses, item-total correlations, and redundancy checks were conducted. Items were removed if they exhibited factor loadings of <0.40, cross-loadings of >0.30, or item-total correlations of <0.30. The best-performing item within sets of highly similar items was retained. This step reduced the pool to 56 items.

Stage 3: Predictive-utility-oriented refinement. Items were further evaluated to ensure suitability for subsequent predictive modeling using (1) correlation with depressive-symptom severity (|ρ| ≥ 0.13), (2) model-based feature importance (≥1%), and (3) multicollinearity control (r ≤ 0.75). The final set consisted of 43 core items retained across the four conceptual domains.

The resulting instrument demonstrated strong psychometric performance, including excellent internal consistency (Cronbach’s α = 0.910), good factor structure (RMSEA = 0.049 and CFI = 0.859), and adequate subdomain coherence, supporting its use as a concise and multidimensional screening tool for child and adolescent mental health.

### Purpose, Structure, and Assessment Domains

The final questionnaire consisted of 50 items, including baseline demographic questions and 43 core items assessing four key domains associated with depression risk: Depression Index: emotional state, cognitive symptoms, motivation, vitality, and psychophysiological responses; Stressful Events: family, school, peer, and environmental stressors experienced in daily life; Screen Use Behaviors: duration and patterns of exposure to different types of electronic screens; and Sleep Status: sleep timing, latency, quality, and daytime functioning.

Recall bias was minimized by having the participants report behaviors for the past week, with morning/afternoon/evening time periods specified to improve accuracy.

### Scoring System and Standardization

All items were scored on a 4-point Likert scale. Positively keyed (protective) items were reverse-coded so that higher scores uniformly indicated greater psychological or behavioral risk. The instrument was organized into four dimensions based on item attributes, with the following scoring rules: Depression Index (Q8–Q20): Q8–Q16 and Q20 scored normally; Q17–Q19 reverse-coded. Stressful Events (Q21–Q34): Higher frequency or impact corresponds to higher scores. Screen Use (Q39–Q43): This dimension was scored according to daily screen exposure duration. Sleep Status (Q45–Q50): Higher scores indicate poorer sleep quality and greater rhythm disruption. Because the dimensions differed in item counts, raw scores were standardized using Z-score normalization:

$$Z=\frac{\text{ Score-Mean }}{\mathrm{SD}},$$

where Mean and SD were derived from the training set to avoid information leakage. Standardized dimension scores were used in model training and feature selection.

For outcome labeling (risk stratification), we used the raw Depression Index total score (Q8–Q20) as the labeling variable. The term *x_j_∈* {1,2,3,4} denotes the response to item *j*. For reverse-coded items, *x^′^_j_*= 5-*x*_j_; otherwise, *x^′^_j_* = *xj*. The Depression Index total score was calculated as

$$S=\sum_{j=Q\,8}^{Q\,20}x^{\prime }\,j.$$

Given 13 items, the theoretical range of* S* is 13–52 (range observed in this dataset: 13–45). Z-score standardization was applied only to predictor (subscale) scores for model training and feature selection, using mean and SD estimated from the training set to avoid information leakage.

### Total Score and Interpretation of Risk Levels

Psychiatric experts at NMHC used empirical score distributions and ICD-11 diagnostic references to establish a four-level risk stratification framework: level 0 (safe): 13.0–28.9, indicating no clinically relevant depressive symptoms; level 1 (mild risk): 29.0–31.9, suggesting mood fluctuations or mild sleep/eating disturbances; level 2 (moderate risk): 32.0–34.9, reflecting persistent negative emotions lasting ≥2 weeks; and level 3 (severe risk): 35.0–52.0 (theoretical); 35.0–45.0 observed in this dataset, indicating pronounced depressive symptoms warranting psychological or medical evaluation. This classification provides clear and operationalized criteria for predictive modeling, school-based screening, and tailored intervention strategies.

### Risk Stratification Logic

A four-level depression risk framework was constructed using raw Depression Index total scores (Q8–Q20) as the labeling variable. The cutoffs (29, 32, and 35) were initially defined by psychiatric experts based on ICD-11/DSM-5 diagnostic guidelines, empirical score distributions, and clinical patterns observed in adolescent cohorts. These expert-defined thresholds were further evaluated using receiver operating characteristic (ROC) curves and Youden index (YI) optimization, which confirmed their discriminative validity. As adolescent depressive risk reflects interacting emotional, behavioral, and environmental factors, the Depression Index total score provides a clinically coherent basis for stratification.

### Model Construction

Utilizing school-based survey data, we employed ML approaches to develop a graded depression risk prediction model. The analytical pipeline comprised the following: (1) data collection, (2) preprocessing, (3) feature engineering, (4) predictive modeling, and (5) comparative evaluation.

Data preprocessing: Following the rigorous application of the inclusion and exclusion criteria, 2202 valid cases remained for analysis (94.5% retention).

Training and testing sets: The dataset was randomly partitioned into training (80%) and testing (20%) subsets. The training set was used for model development and five-fold cross-validation, while the testing set served as an external hold-out dataset for evaluating generalization performance.

Feature selection: A two-stage feature selection framework was implemented, combining hypothesis-driven selection with data-driven statistical filtering to identify the most informative predictors.

Model construction: Three ML algorithms, logistic regression (LR), XGBoost, and CatBoost, were trained and evaluated. Model performance was assessed using ROC analysis and corresponding performance metrics.

### Class Imbalance Handling

Two strategies were used during model training to address the imbalance across the four severity categories (safe 59%, mild 17%, moderate 11%, and severe 13%). Synthetic minority over-sampling technique was used to increase the representation of minority classes (levels 1–3) within the training set, and class-weight adjustment was used in LR, XGBoost, and CatBoost to reduce bias toward the majority class. All balancing methods were applied exclusively to the training set, while the original distribution was maintained in the test set to avoid information leakage.

### Model Evaluation

Calibration curves, partial dependence plots, and SHAP value visualizations were generated to assess performance, analyze feature effects, and evaluate the reliability of probability estimates. Five-fold cross-validation performed on the training set showed that LR exhibited the lowest performance variance and the smallest train-test discrepancy among the three models, supporting its superior generalizability.

### Statistical Analysis

Descriptive statistics were used to summarize demographic characteristics and questionnaire responses. Group differences across the four depression risk levels were examined using chi-square tests for categorical variables and one-way analysis of variance (ANOVA) for continuous variables. Reliability was assessed using Cronbach’s α and the Guttman split-half coefficient. Construct validity was examined using the KMO statistic and Bartlett’s test of sphericity, followed by confirmatory factor analysis (CFA) to evaluate the four-factor structure. Model fit was assessed using the RMSEA, p-close (PCLOSE), the CFI, and related indices. Spearman correlations were computed between all variables and the depression index; variables with |ρ| > 0.13 were retained for subsequent modeling [18, 19]. Two-tailed *p*-values of <0.05 were used for chi-square tests, one-way ANOVA, Bartlett’s test of sphericity, and Spearman correlation analyses. A correlation heatmap was generated to visualize associations between psychological, behavioral, and environmental variables. Cutoff values were examined using ROC curve analysis to establish a statistically robust basis for the four-level depression risk stratification. The 0–3 levels were reformulated into clinically meaningful binary comparisons (e.g., at risk vs. not at risk), and optimal thresholds were identified using the YI. All statistical analyses were two-tailed, with *p* < 0.05 considered statistically significant.

## Results

### Basic Content of the Questionnaire

A total of 2331 questionnaires were collected, of which 2202 valid responses were retained after conducting quality control (94.5% retention rate). The participants were classified into four categories based on depression severity scores: safe (59%), mild (17%), moderate (11%), and severe (13%). Significant differences in mental health scores were observed across screen-based activities and activity interests (all *p* < 0.01; Table 1).

**Table 1.** **Basic information on survey participants**.

Survey item		Depression risk level				*p*-value
		Safe (n = 1299)	Mild depression (n = 380)	Moderate depression risk (n = 237)	Severe depression risk (n = 286)	
Sex, n (%)						0.058
	Male	733 (56.4)	185 (48.7)	128 (54.0)	151 (52.8)	
	Female	566 (43.6)	195 (51.3)	109 (46.0)	135 (47.2)	
Age (y) mean (SD)		12.24 (0.64)	12.24 (0.64)	12.25 (0.63)	12.35 (0.65)	0.446
Watching TV per day, n%						<0.01
	<0.5 h	824 (63.4)	213 (56.1)	111 (46.8)	149 (52.1)	
	0.5–1 h	357 (27.5)	116 (30.5)	87 (36.7)	73 (25.5)	
	1–2 h	94 (7.2)	41 (10.8)	31 (13.1)	39 (13.6)	
	>2 h	24 (1.8)	10 (2.6)	8 (3.4)	25 (8.7)	
Playing games per day, n%						<0.01
	<0.5 h	1005 (77.4)	239 (62.9)	135 (57.0)	168 (58.7)	
	0.5–1 h	198 (15.2)	95 (25.0)	61 (25.7)	67 (23.4)	
	1–2 h	63 (4.8)	36 (9.5)	28 (11.8)	32 (11.2)	
	>2 h	33 (2.5)	10 (2.6)	13 (5.5)	19 (6.6)	
Daily chatting, n%						<0.01
	<0.5 h	1113 (85.7)	295 (77.6)	162 (68.4)	211 (74.2)	
	0.5–1 h	142 (10.9)	65 (17.1)	54 (22.8)	48 (16.8)	
	1–2 h	33 (2.5)	14 (3.7)	15 (6.3)	15 (5.3)	
	>2 h	11 (0.8)	6 (1.6)	6 (2.5)	11 (3.8)	
Learning, per day, n%						<0.01
	<0.5 h	907 (69.8)	234 (61.8)	125 (52.7)	169 (59.1)	
	0.5–1 h	274 (21.1)	89 (23.4)	68 (28.7)	73 (25.5)	
	1–2 h	85 (6.6)	43 (11.3)	31 (13.1)	30 (10.5)	
	>2 h	33 (2.5)	13 (3.4)	13 (5.5)	14 (4.9)	
Scrolling video apps, per day, n%						<0.01
	<0.5 h	1025 (78.9)	246 (64.7)	132 (55.7)	163 (57.0)	
	0.5–1 h	179 (13.8)	86 (22.4)	75 (31.6)	64 (22.7)	
	1–2 h	67 (5.2)	32 (8.4)	19 (8.0)	31 (10.8)	
	>2 h	28 (2.2)	16 (4.2)	11 (4.6)	27 (9.4)	
Activity interest, n (%)						<0.01
	Extreme	228 (17.6)	99 (26.1)	70 (29.5)	108 (37.8)	
	Some	523 (40.2)	168 (44.5)	101 (42.6)	102 (35.7)	
	Occasional	392 (30.3)	79 (20.8)	40 (16.9)	57 (19.9)	
	None	156 (12.0)	33 (8.7)	26 (11.0)	19 (6.6)	

### Questionnaire Reliability and Validity Analysis

Given that the instrument integrated items derived from seven established psychological scales, we first conducted a comprehensive psychometric assessment at the integrated scale level to ensure measurement quality after reconstruction. Reliability analysis demonstrated excellent internal consistency for the full scale (Cronbach’s α = 0.910) and strong split-half reliability (Guttman coefficient = 0.865), indicating high stability and coherence among the integrated items.

Construct validity was evaluated using the KMO measure and Bartlett’s test of sphericity. The integrated questionnaire yielded a KMO value of 0.91, and Bartlett’s test reached statistical significance (*p* < 0.001), confirming adequate sampling and strong inter-item correlations suitable for factor-structure analysis. The CFA results indicated good overall model fit, with an RMSEA of 0.049 (PCLOSE = 0.729) and an acceptable incremental fit index (CFI = 0.859). Because the integrated questionnaire combines items from multiple heterogeneous scales, slightly lower incremental fit indices, such as CFI, are expected in reconstructed multidimensional instruments.

The ROC-YI analysis produced optimal thresholds that closely matched the expert-defined cutoff values of 29, 32, and 35, confirming the statistical consistency and discriminative validity of the four-level classification framework. Collectively, these results demonstrate that the integrated scale possesses solid internal consistency and strong structural validity. All items were standardized to a unified 4-point Likert format to ensure compatibility across the original scales, and score directions were harmonized through reverse-coding when required. Dimension scores and the total score were calculated by summing standardized item scores within their respective domains. This scoring strategy ensured consistency across the integrated dimensions and preserved comparability among items with different original scoring schemes.

### Data Feature Selection Strategy

We implemented a dual-phase feature selection protocol to identify optimal predictor combinations and develop a clinically robust depression risk prediction framework.

Theory-driven selection: Building upon established adolescent mental health research, we systematically identified six core behavioral domains, including (1) screen-based activities and (2) interest-driven activities.

Data-driven selection: We computed Pearson’s correlation coefficients (ρ) between remaining features and depression severity scores. Features exceeding the |ρ| > 0.13 threshold were retained based on effect size magnitude. This procedure yielded clinically relevant predictors, including psychological symptoms and social functioning measures. The resulting multilevel predictive framework fully aligned with the variables listed in **Supplementary Table 1**. The behavioral layer consisted of six features, watching TV, playing games, daily chatting, learning, scrolling video apps, and activity interest, capturing adolescents’ screen-related behaviors and engagement in interest-based activities. The psychological layer consisted of five features: relationships with teachers, concentration, stress levels, perceived family evaluation, and worry about the future, reflecting core emotional, cognitive, and social functioning attributes.

**Fig. 1.**
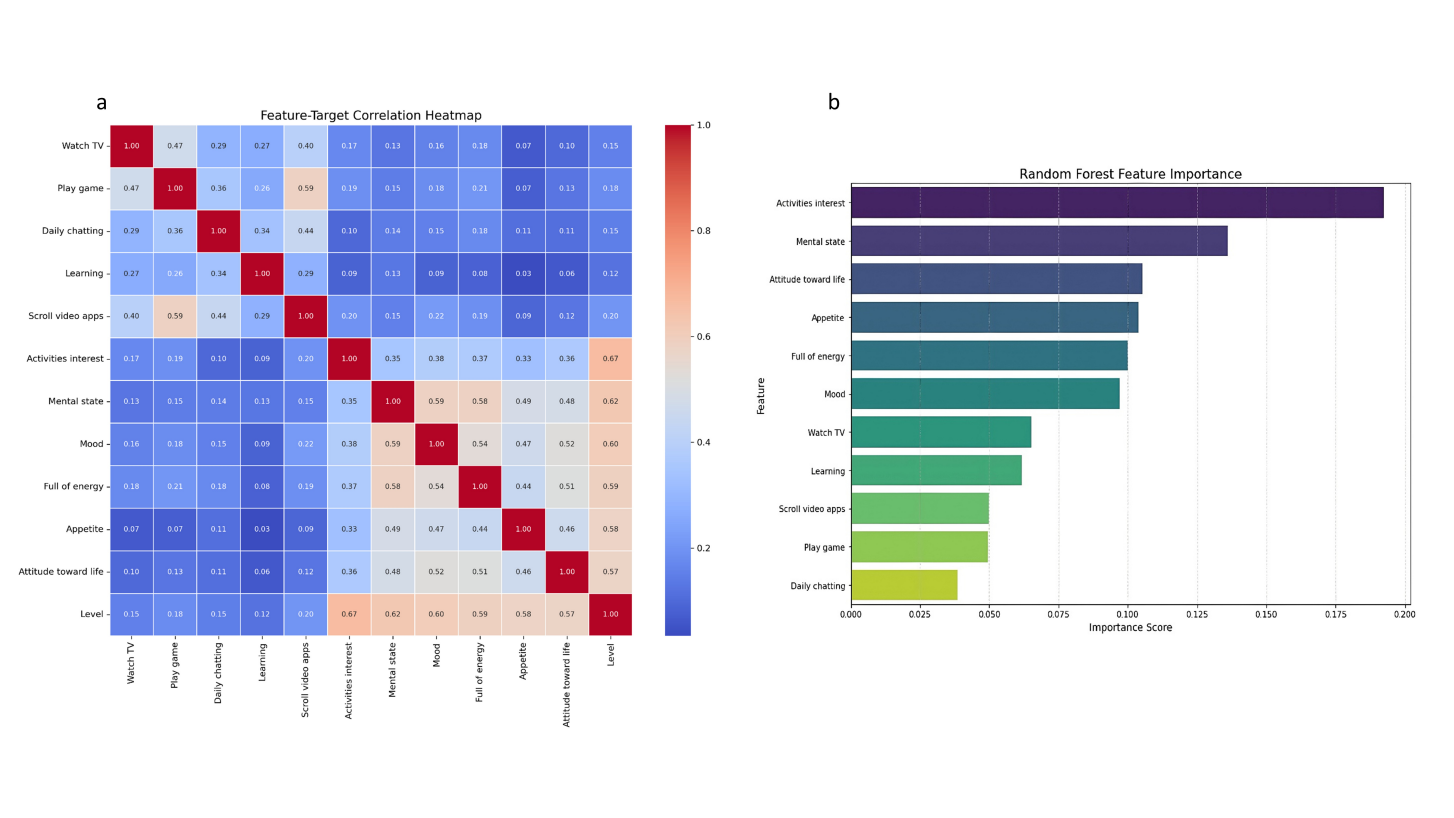
**Feature-target association and importance visualization**. (a) Correlation heatmap depicting feature-target associations (color intensity indicates correlation strength). (b) Random forest–derived feature importance rankings, highlighting activity engagement and psychological functioning as dominant predictors. In this figure, “Level” denotes the severity categories of depressive/anxiety symptoms.

Together, these predictors form a coherent behavior-psychology dual-layer feature framework, offering a mechanistic perspective on how screen use patterns and activity participation contribute to adolescent depression risk. The operational definitions of these variables are provided in **Supplementary Table 1**. Both the correlation matrix and the random forest feature-importance ranking further supported this feature-selection framework, consistently highlighting activity interest (importance weight = 22.5%) and psychological-function features (e.g., psychological status, 18.3%) as the primary predictors (Fig. 1).

### Model Construction

Given the multifactorial nature of adolescent depression risk prediction, we employed three distinct ML approaches for systematic comparison, ranging from interpretable linear models to advanced ensemble methods, to optimally balance predictive performance with clinical interpretability.

### Construction of the Three Models

Rigorous feature selection identified 11 clinically relevant predictors as model inputs. LR, XGBoost, and CatBoost were then implemented to allow a comparative analysis of algorithmic performance in predicting clinical risk.

#### LR Model

The LR model applies a sigmoidal transformation to linear combinations of predictors, yielding interpretable probabilities in which coefficient signs directly reflect directional effects: positive coefficients (e.g., play game) indicate increased risk, and negative coefficients (e.g., activity diversity) indicate reduced risk.

**Fig. 2.**
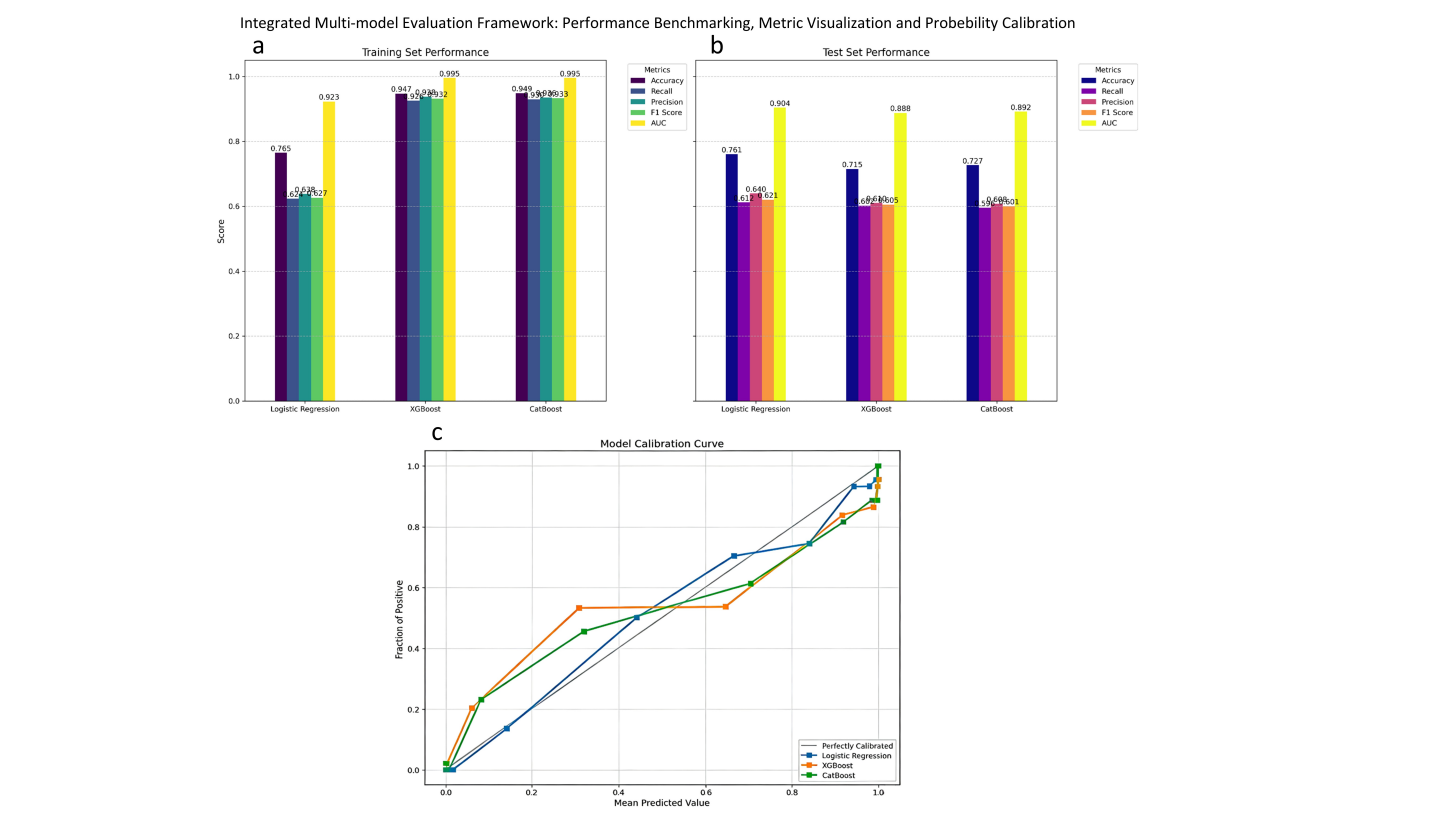
**Performance and calibration of three depression risk prediction models**. (a) Training and test results. XGBoost achieved the highest test accuracy (0.918), CatBoost obtained the highest recall (0.915), and LR showed the smallest train–test discrepancy. (b) Multidimensional comparison of model performance. XGBoost showed balanced indicators, while LR showed greater variability. (c) Calibration curves. XGBoost was closest to the ideal reference line. CatBoost tended to underestimate probabilities near the 0.5 threshold. XGBoost, extreme gradient boosting; CatBoost, categorical boosting.

L2 regularization was applied to mitigate overfitting. This parsimonious model provided a stable baseline for comparing more complex models. XGBoost achieved the highest training accuracy but displayed a clear train-test performance gap indicative of overfitting (Fig. 2). CatBoost showed balanced performance across several metrics, whereas LR maintained a more consistent performance between the training and test sets. Importantly, LR also exhibited the smallest discrepancy across the five-fold cross-validation, further supporting its superior generalizability and robustness.

#### CatBoost Model

The assessment instruments included several categorical and ordinal variables (e.g., activity engagement levels and psychological symptom scores). Traditional models require these variables to be converted through one-hot encoding. CatBoost’s processes categorical and ordinal data directly using its ordered boosting strategy, allowing these measures to be processed without losing their original structure. This approach helps preserves hierarchical relationships within psychological constructs.

#### XGBoost Model

XGBoost uses an iterative residual-based learning process that can capture nonlinear relationships between predictors. This capability is useful for identifying complex risk patterns, such as the combined effect of high screen exposure and low activity engagement, which are often missed by linear models.

### Feature Engineering and Data Standardization

**Fig. 3.**
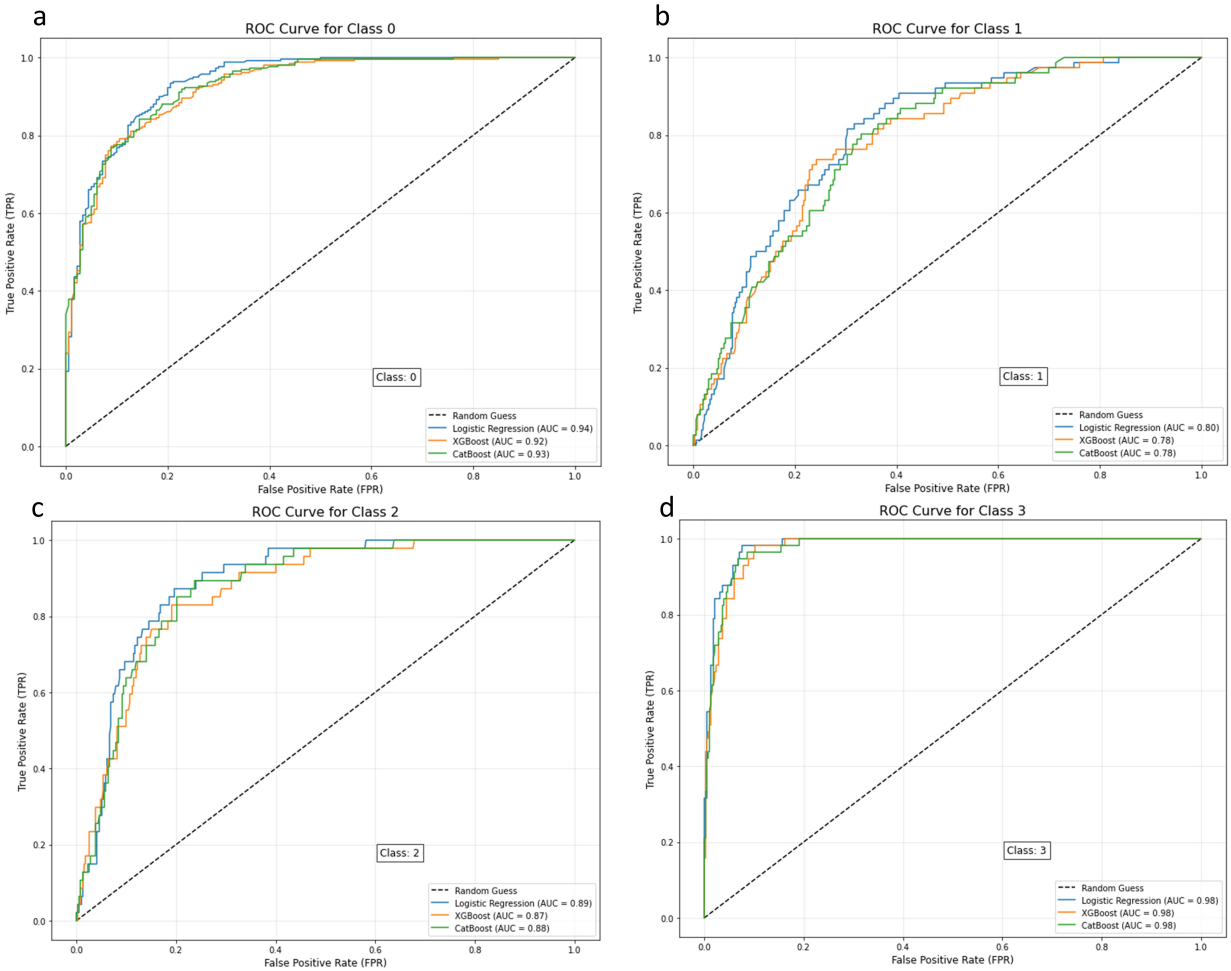
**Comparative ROC analysis of multiclass depression risk classifiers (classes 0–3, one-vs.-rest)**. (a) Class 0: All models achieved a high AUC (0.92–0.94), with LR being slight higher. (b) Class 1: LR achieved the highest AUC (0.99), whereas XGBoost and CatBoost showed lower performances (AUC = 0.78–0.79). (c) Class 2: All models performed well (AUC = 0.87–0.89), with LR being the highest. (d) Class 3: All models showed near-perfect performance (AUC ≥0.98). Class labels: 0 = safe, 1 = mild risk, 2 = moderate risk, and 3 = severe risk. ROC, receiver operating characteristic; AUC, area under the curve; LR, logistic regression; XGBoost, extreme gradient boosting; CatBoost, categorical boosting.

Given substantial feature scale variation (e.g., hourly screen time vs. 4-point Likert-scale psychological measures), we standardized all features using z-scoring by transforming them to an N (0, 1) distribution. This normalization enhances convergence in gradient-based algorithms (e.g., LR) while enabling comparable feature importance metrics (e.g., SHAP values) across variables. Both CatBoost and LR maintained well-calibrated probability predictions, as evidenced by their close approximation to the ideal calibration curve (Fig. 3).

We employed an 80:20 train-test split with stratified sampling preserving original risk-level distributions for data partitioning. Standardization parameters were derived exclusively from the training set. They were then applied to the test data to prevent data leakage.

### Model Evaluation and Analysis

This study systematically examined the predictive patterns of screen time and activity interest in adolescent depression risk using SHAP value interpretation, partial dependence analysis, and comparative modeling approaches. ROC curves were used to compare the performance of the three models in the four-class classification task (Fig. 3). LR displayed the highest area under the curve (AUC) for categories 0 and 3, demonstrating its precision in identifying extreme states.

### Direct Effect of Screen Time on Depression Risk Levels

**Fig. 4.**
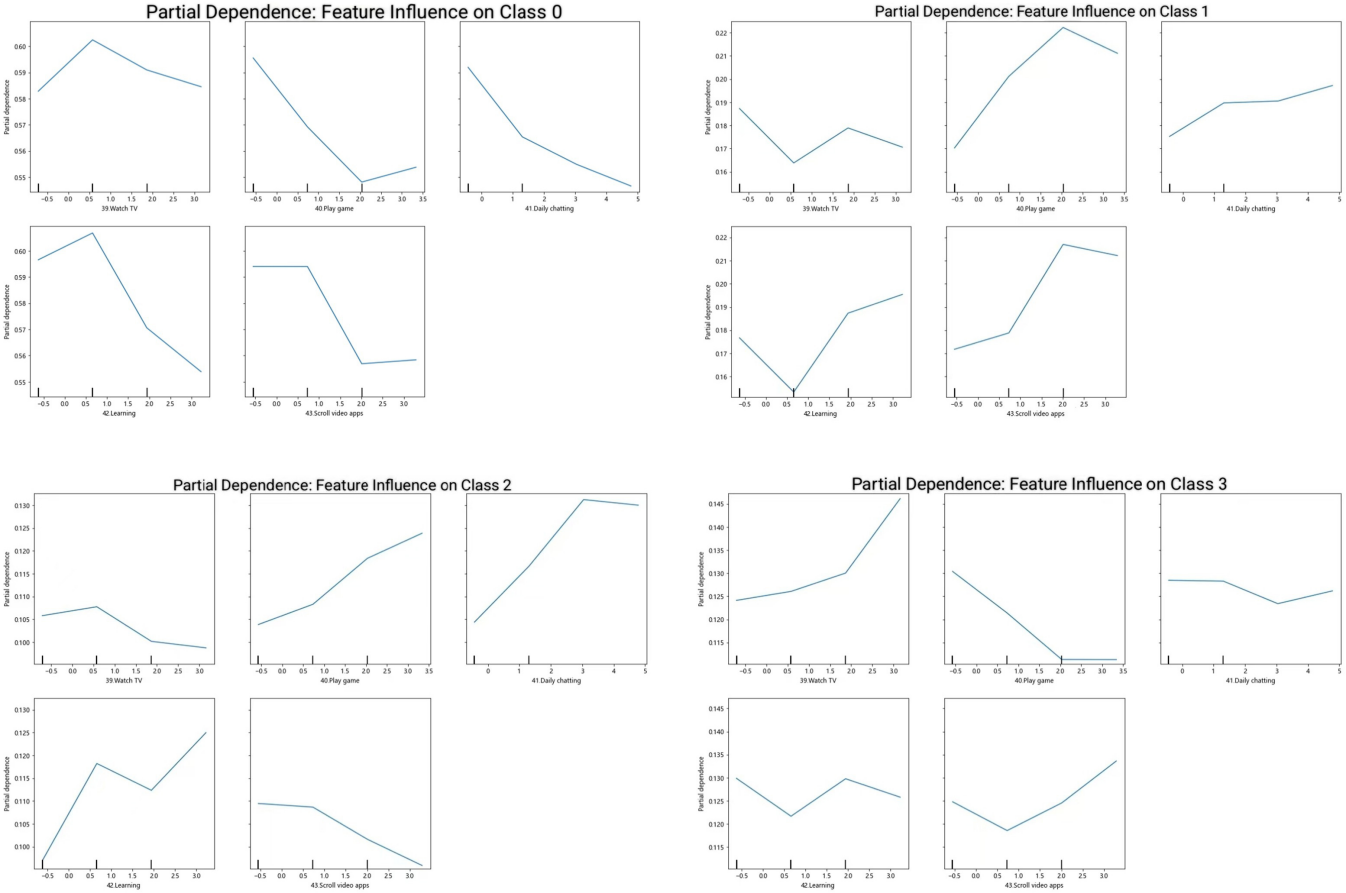
**Partial dependence plots illustrating the effects of screen time behaviors on four depression risk categories (0–3)**. The plots reveal how behaviors such as watching TV and playing games influence depression risk levels. All models demonstrated strong discriminative capacity (AUC >0.80). LR and CatBoost exhibited particularly stable nonlinear response patterns. AUC, area under the curve.

SHAP analysis revealed nonlinear associations between screen-related behaviors and depression risk levels. Watching TV, playing games, scrolling video apps, daily chatting, and learning consistently exhibited positive SHAP values, indicating positive associations with elevated depression risk. Playing games and watching TV showed particularly strong effects in the high-risk category (category 3). Partial dependence analysis uncovered differential risk patterns. Screen time exhibited varying risk associations across activities. Nonlinear predictive relationships were found between specific screen behaviors and graded depression risk levels (Fig. 4). Moderate TV watching demonstrated protective associations in low-risk individuals (category 0). In contrast, excessive game playing and scrolling video apps beyond optimal thresholds showed dose-dependent risk elevation. Notably, both insufficient and excessive game playing exhibited U-shaped associations with high-risk depression.

The beeswarm plot visualization corroborated these nonlinear patterns. It showed risk-level-dependent variations in feature effects, with feature value magnitudes directly modulating their directional influence on risk prediction. The partial dependence analysis showed nonlinear relationships between specific screen behaviors and graded depression risk (Fig. 4). It also highlighted critical transition thresholds during excessive usage.

### Relationship Between Activity Interest and Different Depression Risk Levels

**Fig. 5.**
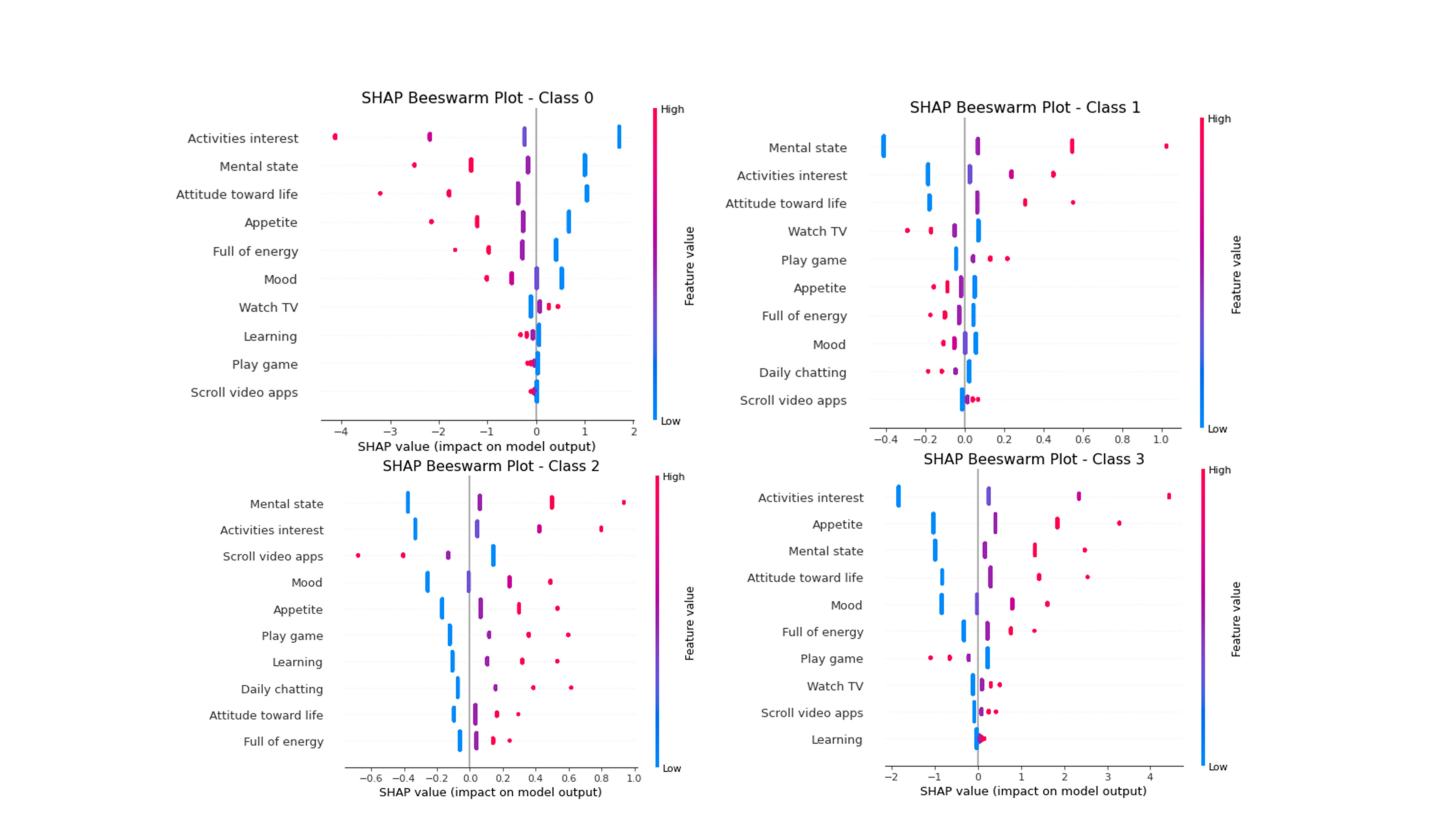
**SHAP beeswarm plot illustrating multivariate feature impacts in four depression risk categories (0–3)**. Each point represents a feature’s contribution to individual predictions, with SHAP values indicating the impact direction and magnitude and color denoting feature intensity. For clarity, only the top 10 features are presented. Although the full model included 11 variables, the omitted feature had a minimal contribution and did not affect the interpretation (see **Supplementary Table 2** for specific data). SHAP, shapley additive explanations.

SHAP value rankings revealed activity interest as a consistently top-ranked feature, particularly for extreme-risk classifications (categories 0 and 3). Partial dependence analysis identified an inverted U-shaped relationship. While moderate activity engagement optimally reduced risk, both excessive participation and its complete absence decreased its protective benefits. This indicated dose-dependent protective effects of activity engagement. In high-risk individuals (category 3), activity interest moderated the adverse effects of screen time, potentially through emotion regulation and social support enhancement. Fig. 5 presents a beeswarm plot quantifying both directionality and magnitude of feature contributions to model predictions. The results confirmed the dominant predictive importance of activity engagement and psychological factors.

### Interaction Mechanism Between Screen Time and Activity Interest

**Fig. 6.**
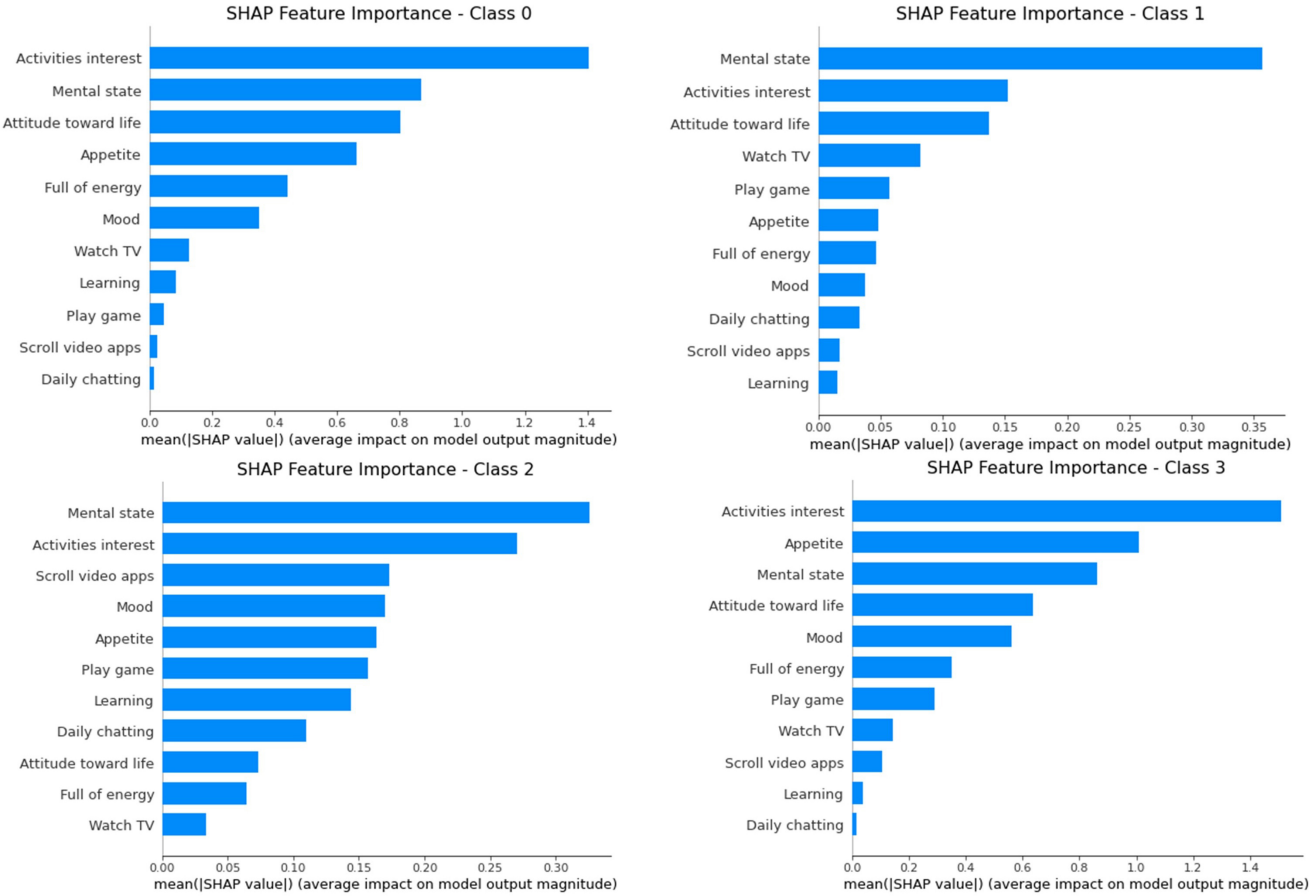
**SHAP feature importance plot based on mean absolute SHAP values in different depression risk categories (0–3)**. The X-axis shows each feature’s average impact on model output. Higher activity-interest values with positive SHAP scores strongly predicted classification into category 0. The varying feature importance rankings illustrate the model’s ability to distinguish risk levels, with psychological state (e.g., appetite SHAP: class 0 = 0.850, class 1 = 0.309, class 2 = 0.178, and class 3 = 0.980) and screen-related behaviors showing category-specific influence patterns (**Supplementary Table 2**). SHAP, shapley additive explanations.

Model comparisons revealed a clear competition between screen time and activity engagement. Increased screen use consistently displaced opportunities for interest-based activities. This effect was most evident in moderate- and high-risk individuals. Conversely, limited screen exposure coupled with diversified activity participation was associated with stable low-risk profiles. Partial dependence analysis confirmed this behavioral divergence. The models showed strong predictive validity for these interactions (F1 >0.78; maximum AUC = 0.98). Fig. 6 displays the ranked SHAP values across risk categories, demonstrating category-specific variation and the model’s capacity for differentiated risk assessment.

### Intervention Insights and Model Value

Our findings suggest that adolescent depression prevention should follow a principle of moderation and balance.

For low-risk individuals (category 0), interventions should maintain current healthy patterns. This includes balancing screen exposure (e.g., watching TV ≤1 hour/day) alongside sustained engagement in interest-based activities. The LR model showed strong discriminative validity in this subgroup (AUC = 0.93), supporting its utility for population-level screening and progress monitoring.

High-risk individuals (category 3) require more intensive strategies. These include stricter control of excessive screen exposure, especially prolonged TV watching and repeatedly scrolling video apps, through the structured promotion of social interaction and physical activity. The LR model achieved exceptional performance in this subgroup (AUC = 0.98), enabling precise case identification and effective follow-up.

High-AUC models provide enhanced capability for detecting behavior-behavior interactions and predicting transitions across risk levels. These strengths support personalized behavior tracking and targeted intervention planning.

## Discussion

This study adopted a multi-school, regional cross-sectional survey design by randomly selecting two primary schools and two junior high schools in Chongchuan District, Nantong City. Theory-driven and data-driven approaches were integrated to develop ML models for predicting depression risk in adolescents. Comprehensive model evaluation revealed that LR achieved optimal overall performance, whereas CatBoost exhibited the most balanced recall-precision trade-off, confirming that screen time and activity interest are key factors in assessing depression risk.

An integrated questionnaire was reconstructed from seven established scales, creating a multidimensional structure that naturally increased model complexity. This structural heterogeneity can influence incremental fit indices, and such patterns have been documented in integrated psychological instruments [20]. Importantly, the strong RMSEA and PCLOSE values in this study indicate that the four-factor structure adequately captured the primary domains relevant to adolescent depression. Future refinement, such as item-level revision and multi-group validation across developmental stages, will further clarify structural stability and ensure that the instrument maintains robust measurement properties in broader applications [21].

Excessive screen time among adolescents may adversely affect mental health and elevate depression risk. Pre-sleep exposure to screen-emitted BL suppresses melatonin secretion and disrupts circadian rhythms. This disruption leads to delayed sleep and reduced sleep efficiency, physiological disturbances associated with greater depression vulnerability [5]. Our findings align with this pattern, as higher depression levels were predicted in adolescents with longer evening screen time. Additionally, sustained digital stimulation from entertainment-based content may dysregulate the dopamine reward system, contributing to anhedonia, a core feature of depression [22].

Beyond these neurobiological pathways, the present findings can also be interpreted within established psychiatric frameworks [23]. According to DSM-5 diagnostic criteria, core depressive symptoms in youth, such as anhedonia, sleep–wake disturbances, irritability, and impaired concentration [24], closely align with the behavioral patterns identified in this study. Evening screen exposure is consistent with sleep disruption as a transdiagnostic pathway, commonly co-occurring with anxiety and depressive symptoms in youth [25]. Likewise, excessive or dysregulated screen use resembles the maladaptive behavioral patterns seen in problematic internet use, a condition frequently accompanied by attentional and emotion-regulation difficulties [26].

The results further provide implications for psychiatric intervention. Activity interest, identified as the strongest protective factor in our SHAP analysis (mean absolute SHAP for class 0 = 1.580 and class 3 = 1.550), overlaps substantially with the core components of cognitive-behavioral therapy (CBT), particularly behavioral activation [27]. Enhancing interest-based activities may counteract reward-system dysregulation and decrease anhedonia. In contrast, excessive screen use may be conceptualized within CBT formulations as avoidance behavior, supporting modules such as stimulus control and sleep-hygiene training [28].

Taken together, integrating ML-derived behavioral markers with DSM-5 symptom domains [29] offers a more comprehensive framework for understanding depression risk in youth [30]. This integrated perspective also supports clinical practices in child and adolescent psychiatry, including structured assessment, early identification, and stepped-care interventions.

The study also identified significant interaction effects between screen time and activity interest. This risk-protection interplay establishes a theoretical framework for designing personalized interventions. Enhancing activity interest may mitigate depression progression in individuals with high screen times [31]. This suggests a synergistic behavioral-psychological protective pathway [32]. The protective effect may operate by modulating neurotransmitter systems, such as dopamine and serotonin, which are crucial for maintaining mood stability [33]. A key contribution of this study is the identification of a significant moderating effect of activity interests. High levels of activity engagement buffered the negative impact of excessive screen time and showed a dose-dependent protective pattern. This behavioral-psychological interaction provides a mechanistic explanation for why activity engagement remains a central determinant of depression resilience among adolescents, supporting targeted intervention strategies that simultaneously reduce excessive screen exposure and strengthen interest-based activities.

Previous research demonstrated that ML techniques could effectively identify depression and anxiety risk factors, supporting mental health interventions [34]. Consistent with these findings, our study showed that ML models could predict adolescent depression risk by incorporating screen time and activity interest features. Mardini *et al*. [35] used XGBoost to identify depression and anxiety in adolescents. Qirtas *et al*. [36] applied LR and other algorithms to predict loneliness and depression using screen time-related features. The predictive accuracy of the model in this study further validates the effectiveness of these features in adolescent depression risk assessment.

ML models, particularly when used in real-time monitoring, offer a cost-effective, time-efficient approach for early intervention, especially in schools and resource-limited settings [37]. The system requires minimal input data, reduces stigma concerns, and enhances applicability across diverse environments [38]. The findings emphasize the importance of screen time regulation (e.g., usage limits and quality content engagement [39]) and interest development (e.g., participation in school clubs [40] and family-child activities [41]). Addressing both behavioral and psychological factors may effectively reduce depression risk [42].

While this study advances the understanding of adolescent depression risk assessment, several limitations should be noted. First, the comparisons presented in Table 1 are intended as descriptive summaries of behavioral patterns within each depression-risk category rather than inferential tests of independent group differences. Because screen time and activity interest items contributed to the total score used for risk stratification, direct statistical comparisons may introduce partial circularity. These findings should be interpreted as contextual characterization rather than evidence of independent associations. Importantly, the ML feature selection and SHAP analyses, which do not rely on total-score-based grouping, avoid this limitation. Second, reliance on self-reported measures may introduce response biases in reporting screen time and activity interest. The scale used to measure activity interest (extreme/some/occasional/none) did not differentiate activity types (e.g., physical, artistic, or social activities). This limitation may mask their differential effects on depression risk. Future research should distinguish activity types to better understand their specific impacts on adolescent mental health. Finally, the cross-sectional design prevents causal inference. Thus, whether excessive screen use contributes to depression or whether depressive symptoms increase screen use as a coping mechanism remains unclear. Future longitudinal studies should examine these dynamic mechanisms and explore the bidirectional relationship between screen time and depression.

Future work will focus on developing targeted interventions for adolescent mental health and investigating the applications of ML in mental health assessment and intervention.

## Conclusions

This study demonstrated that ML could substantially improve the efficiency of identifying adolescents at risk for depression by integrating information on screen time and activity interests. The developed model serves as a practical early-stage screening tool, capable of supporting timely detection and intervention in school settings.

Although the model shows strong potential, it is intended for screening rather than diagnostic purposes, and further validation in clinical contexts is needed. In practice, embedding this model in schools could help teachers and parents monitor students’ mental health more proactively, enabling early support for those who may be struggling. This model provides a rapid, low-cost, and scalable approach to mental-health monitoring and offers meaningful value for promoting healthier psychological development in adolescents.

## Availability of Data and Materials

The inquiries of original contributions presented in the study can be directed to the corresponding authors.
