# Supplementary material for: Impact of the Interaction Between Screen Time and Activity Interests on Adolescent Depression Risk: Construction of a Predictive Model Based on Machine Learning
Source: Actas Esp Psiquiatr. 2026 Apr 15;54(2):500–15. doi: 10.62641/aep.v54i2.2068 (PMC13180681; doi:10.62641/aep.v54i2.2068)
Supplement: Supplementary file 1 [file ActEsp-54-2-500-515-s1.zip › Supplementary Material.pdf]

Supplementary Material 1：

Child and Adolescent Mental Health Screening Questionnaire

Informed Consent Statement

Dear classmates,

Hello! Thank you very much for participating in this psychological questionnaire survey. The questionnaire is divided into two parts: "Baseline Information Collection" and "Core Scale Measurement". Please read each item carefully and answer truthfully according to your actual situation. There are no right or wrong answers.

Participation in this survey is completely voluntary. You may refuse to answer any question or withdraw at any time without any negative consequences. The survey is anonymous, and all responses will be kept strictly confidential and used only for research purposes.

By proceeding with the questionnaire, you indicate that you have understood the above information and agree to participate.

We sincerely appreciate your cooperation and wish you academic progress and a pleasant life!

Part 1: Baseline Information Collection (Independent Module)

(Note: This part collects basic background information of participants for statistical analysis only and is not included in the core measurement dimensions of the scale)

1. Student Identification Number (School + Grade + Class + Student ID):
2. Age (in years):
3. Gender:

   A. Male B. Female

4. Height (centimeters):
5. Weight (kilograms):
6. Number of cohabiting family members (persons):

7. Family Relationship: (　　　)

A. One-child families　　B. Multi-child families

Part 2: Core Scale Measurement (4 Defined Core Dimensions)

(Note: This part is the core measurement items of the scale, using a 4-point Likert scale to assess key psychological and behavioral characteristics related to depressive risk

I.Please provide a summary of your academic progress and personal life over the past two weeks.

8. How have you been feeling recently? (　　　)

A. Very good　B. Good　C. Average　D. Poor

9. Are you interested in participating in activities (such as reading books, sports activities, etc.)? (　　　)

A. Very interested　　　　B. Sometimes interested

C. Occasionally interested　　D. Not interested

10. How has your sleep been recently? (　　　)

A. Sleeping very well　　B. Sleeping well

C. Sleeping little　　　D. Can't sleep

11. What is your current mental state? Do you feel energetic or fatigued? (　　　)

A. Full of energy　　B. Energetic　　C. Occasionally fatigued　　D. Fatigued

12. How has your appetite been recently? (　　　)

A. Very good　　B. Good　　C. Average　　D. Poor

13. How do you perceive your family's evaluation of you? (　　　)

A. Outstanding　　B. Excellent　　C. Ordinary　　D. Bad

14. Can you maintain focus during tasks such as reading or attending classes? (　　　)

A. Very focused　　B. Focused　　C. Occasionally distracted　　D. Not focused

15. How fast do you act or speak in daily life? (　　　)

A. Very fast　　B. Fast　　C. Normal　　D. Slow

16. What is your attitude toward life? (　　　)

A. Very optimistic　B. Optimistic　C. Indifferent　D. Pessimistic

17. How do you handle situations, such as feeling nervous before an exam? (　　　)

A. Calm　B. Indifferent　C. Nervous　D. Very nervous

18. Do you overthink before making decisions, such as before signing up for a competition? (　　　)

A. Often　B. Sometimes　C. Occasionally　D. Never

19. Do you worry in advance about things that haven't happened yet? (　　　)

A. Never　B. Occasionally　C. Sometimes　D. Often

20. Do you stay excited for a long time after experiencing something happy? (　　　)

A. Often　B. Sometimes　C. Occasionally　D. Never

21. Have you ever been misunderstood or wrongly blamed? (　　　)

A. Never　B. Occasionally　C. Sometimes　D. Often

22. Do you have a good relationship with your classmates? (　　　)

A. Very good　B. Good　C. Average　D. Bad

23. Have your living habits (diet, rest, etc.) changed significantly? (　　　)

A. No　B. Occasionally　C. Sometimes　D. Often

24. How do you feel about attending school? (　　　)

A. Enthusiastic　B. Positive　C. Neutral　D. Negative

25. Do your parents spend time with you often? (　　　)

A. Often　B. Sometimes　C. Occasionally　D. Never

26. Do you wish your parents spent more time with you? (　　　)

A. Often　B. Sometimes　C. Occasionally　D. Not at all

27. Do you have a good relationship with your teachers? (　　　)

A. Very good　B. Good　C. Average　D. Bad

28. Have you ever embarrassed yourself in public? (　　　)

A. Never　B. Occasionally　C. Sometimes　D. Often

29. Do you think your living conditions are good? (　　　)

A. Very good　B. Good　C. Average　D. Poor

30. How do you feel about your family's relationship? (　　　)

A. Very good B. Good C. Average D. Poor

31. How do you view the anticipated selection (such as the "Three Merit Students")? ( )

A. Definitely eligible B. Hoping to be eligible

C. Possibly eligible D. Definitely ineligible

32. Have you ever been criticized? ( )

A. Never B. Occasionally C. Sometimes D. Often

33. Have you ever transferred schools or taken a break from school? ( )

A. Never B. Once C. Twice D. Three or more times

34. Have you ever been in a fight? ( )

A. Never B. Occasionally C. Sometimes D. Often

35. Do you feel stressed about your current life? ( )

A. Never B. Occasionally C. Sometimes D. Often

36. Where does your stress mainly come from? (If none, select A) ( )

A. No stress B. School or peers

C. Family or relatives D. Society or environment

37. Do you think exercise benefits brain development? ( )

A. Yes B. No effect C. No D. Don't know

38. How do you think parents should help build your self-confidence? ( )

A. Frequent praise B. Occasional praise

C. Occasional criticism D. More criticism

II. During the past two weeks, what were the average durations of your screen time on electronic products and physical exercise?

39. How much time do you spend watching TV daily? ( )

A. Less than 0.5 hours B. 0.5-1 hour C. 1-2 hours D. More than 2 hours

40. How much time do you spend playing video games daily? ( )

A. Less than 0.5 hours B. 0.5-1 hour C. 1-2 hours D. More than 2 hours

41. How much time do you spend chatting (using WeChat, QQ, etc.) daily? ( )

A. Less than 0.5 hours B. 0.5-1 hour C. 1-2 hours D. More than 2 hours

42. How much time do you spend on homework or online courses (using phone, tablet,

etc.) daily? (　　　)

A. Less than 0.5 hours　B. 0.5-1 hour　C. 1-2 hours　D. More than 2 hours

43. How much time do you spend on entertainment video apps (Tiktok, Kuaishou, Bilibili, etc.) daily? (　　　)

A. Less than 0.5 hours　B. 0.5-1 hour　C. 1-2 hours　D. More than 2 hours

44. How much time do you spend on physical exercise daily (including running, recess exercises, ball games, and outdoor activities)? (　　　)

A. Less than 0.5 hours　B. 0.5-1 hour　C. 1-2 hours　D. More than 2 hours

III. How has your sleep been in the recent month?

45. What time do you usually go to bed at night? (　　　)

A. 8:00 PM or earlier　B. Around 9:00 PM

C. Around 10:00 PM　D. 11:00 PM or later

46. How long does it usually take for you to fall asleep after going to bed? (　　　)

A. Within 15 minutes　B. Around 30 minutes

C. Around 60 minutes　D. More than 90 minutes

47. What time do you usually wake up in the morning? (　　　)

A. 5:00 AM or earlier　B. Around 6:00 AM

C. Around 7:00 AM　D. 8:00 AM or later

48. How would you rate your sleep quality? (　　　)

A. Very good　B. Good　C. Average　D. Poor

49. Do you feel sleepy during the day? (　　　)

A. Not at all　B. Sometimes　C. Occasionally　D. Very sleepy

50. How is your energy level during the day when doing tasks? (　　　)

A. Excellent　B. Good　C. Moderate　D. Poor

The survey ends here, thank you for your cooperation!

Supplementary Table S1. Abbreviations and Definitions of Key Feature Variables

Variables	Detailed explanation
Watch TV	Watch TV daily
Play game	Play video games daily
Daily chatting (per day)	Daily Chatting (using WeChat, QQ, or other messaging apps)
Learning	Daily checking of homework, online courses, etc. (using mobile phones, tablets, or other electronic devices)
Scroll video apps	Daily use of video entertainment apps like Douyin, Kuaishou, Bilibili, etc
Activities interest	Are you interested in participating in activities (such as reading books, sports activities, etc
Relationship with teachers	Do you have a good relationship with your teachers
Maintain focus	Can you maintain focus, for example, when reading books or during class
Feel stressed	Do you feel stressed in your current life
Family’s evaluation	How do you think your family evaluates you
Worry about future	Do you worry about things that haven't happened yet

Supplementary Table S2. SHAP Feature Importance Values Across Depression Risk Categories (Mean Absolute SHAP Values)

Feature	Category 0 (Safe)	Category 1 (Mild)	Category 2 (Moderate)	Category 3 (Severe)
Activities interest	1.580120	0.340737	0.371257	1.549600
Mental state	0.944687	0.158214	0.213631	0.889270
Appetite	0.849901	0.308959	0.178418	0.980442
Attitude toward life	0.847066	0.115936	0.238585	0.724417
Full of energy	0.482353	0.093802	0.169716	0.406439
Mood	0.475508	0.196684	0.130434	0.541758
Learning	0.111462	0.057419	0.094558	0.074323
Watch TV	0.057282	0.088465	—	0.077917
Daily chatting	0.027801	0.075201	0.085046	—
Play game	0.016118	0.065359	0.109914	0.191391
Scroll video apps	—	—	0.115436	0.056782
